# Diffusion Magnetic Resonance Imaging: What Water Tells Us about Biological Tissues

**DOI:** 10.1371/journal.pbio.1002203

**Published:** 2015-07-23

**Authors:** Denis Le Bihan, Mami Iima

**Affiliations:** 1 NeuroSpin, Bâtiment 145, CEA Saclay-Center, Gif-sur-Yvette, France; 2 Human Brain Research Center, Kyoto University Graduate School of Medicine, Kyoto, Japan; 3 Department of Diagnostic Imaging and Nuclear Medicine, Kyoto University Graduate School of Medicine, Kyoto, Japan; 4 The Hakubi Center for Advanced Research, Kyoto University, Kyoto, Japan

## Abstract

Since its introduction in the mid-1980s, diffusion magnetic resonance imaging (MRI), which measures the random motion of water molecules in tissues, revealing their microarchitecture, has become a pillar of modern neuroimaging. Its main clinical domain has been the diagnosis of acute brain stroke and neurogical disorders, but it is also used in the body for the detection and management of cancer lesions. It can also produce stunning maps of white matter tracks in the brain, with the potential to aid in the understanding of some psychiatric disorders. However, in order to exploit fully the potential of this method, a deeper understanding of the mechanisms that govern the diffusion of water in tissues is needed.

## Abstract 2

This Essay looks at the history and future potential of diffusion magnetic resonance imaging (MRI), a powerful imaging concept that gives insight into brain architecture and function—whether normal or diseased—completely noninvasively.

In the mid-1980s, we showed that water diffusion in the human brain could be imaged by using magnetic resonance imaging (MRI) [1]. Since then, diffusion MRI has enjoyed a dramatic growth, with about 24,000 articles referenced in PubMed in 2014. MRI is a medical imaging technique consisting of magnetizing body atom nuclei, generally hydrogen nuclei of water molecules, using a very strong magnetic field (typically 30,000 to 60,000 times the earth’s natural magnetic field). The resulting very tiny magnetization can be manipulated in time by sending radiofrequency wave pulses at a resonant frequency. In turn, magnetized nuclei re-emit radiofrequency waves, creating a signal that is received through a coil (a kind of antenna), giving information on the nuclei magnetization properties. Magnetic field “gradient” pulses are used in addition to induce small variations of the magnetic field (and the associated radiowaves’ resonant frequency) in space, so as to spatially encode the magnetization information and create images. Magnetization varies a lot between tissues and various disease conditions, making MRI a very versatile imaging modality. However, the resolution of MRI images used for clinical practice often remains limited, typically around 1 mm (microscopic MRI is possible, but with dedicated preclinical MRI systems using ultra-high magnetic fields; see below). The concept of diffusion MRI emerged as a way to probe tissue structure at a microscopic (invisible) scale, although images are acquired at a millimetric scale: during their random, diffusion-driven displacements in the tissue, the water molecules probe the tissue structure at a microscopic scale, interacting with cell membranes, thus providing unique information on the functional architecture of tissues. Diffusion MRI has become a pillar of modern clinical imaging, used mainly to investigate neurological disorders such as acute brain ischemia, although it is now also a standard imaging method for other organs too, especially for the management of cancer patients. Indeed, diffusion MRI that does not require any tracer injection is rapidly becoming a modality of choice to detect and characterize malignant lesions. Moreover, in the brain, diffusion anisotropy in white matter can be exploited to produce stunning three-dimensional maps of brain connections, revealing faulty connections in some psychiatric disorders. More recently, diffusion MRI has been applied to monitor the dynamic changes occurring in the neural tissue structure during activation, a new approach to investigate functional neuroimaging and the mechanisms underlying neuronal activation.

It is amazing that all these applications of diffusion MRI have emerged or developed while so little is known about water diffusion mechanisms in biological tissues. The relative importance of many factors governing water in tissues and their effects on the observed MRI signal are still not fully understood and are sometimes a subject of controversy.

We will discuss the main applications and the outstanding issues remaining in the field in more detail below.

## Principles of Diffusion MRI and Key Concepts

The botanist Robert Brown observed in 1827 through a microscope that pollen grains suspended in water move spontaneously in a random manner, but he was not able to determine the mechanisms that caused this motion. Independently, the macroscopic phenomenon of “diffusion,” referring to the net movement of a substance from a region of high concentration to a region of low concentration, was described mathematically by Adolf Fick in 1855. Unexpectedly, diffusion (a visible phenomenon) was linked by Einstein to Brownian motion in the context of the theory of heat to prove the existence of (invisible) atoms and molecules [1]. Einstein showed how Brownian motion resulted from particles being moved by individual molecules and how their displacement was linked to the diffusion coefficient of Fick’s laws, thus bridging for the first time the concepts of diffusion and Brownian motion. Based on this explanation of the Brownian motion, Jean Perrin was able to experimentally determine the size of the water molecule from diffusion measurements in 1908. With diffusion MRI, one usually investigates the self-diffusion of water molecules in tissue water. (Diffusion of other molecular moieties, such as some metabolites or neurotransmitters, can also be studied with MR spectroscopy.)

**Fig 1:**
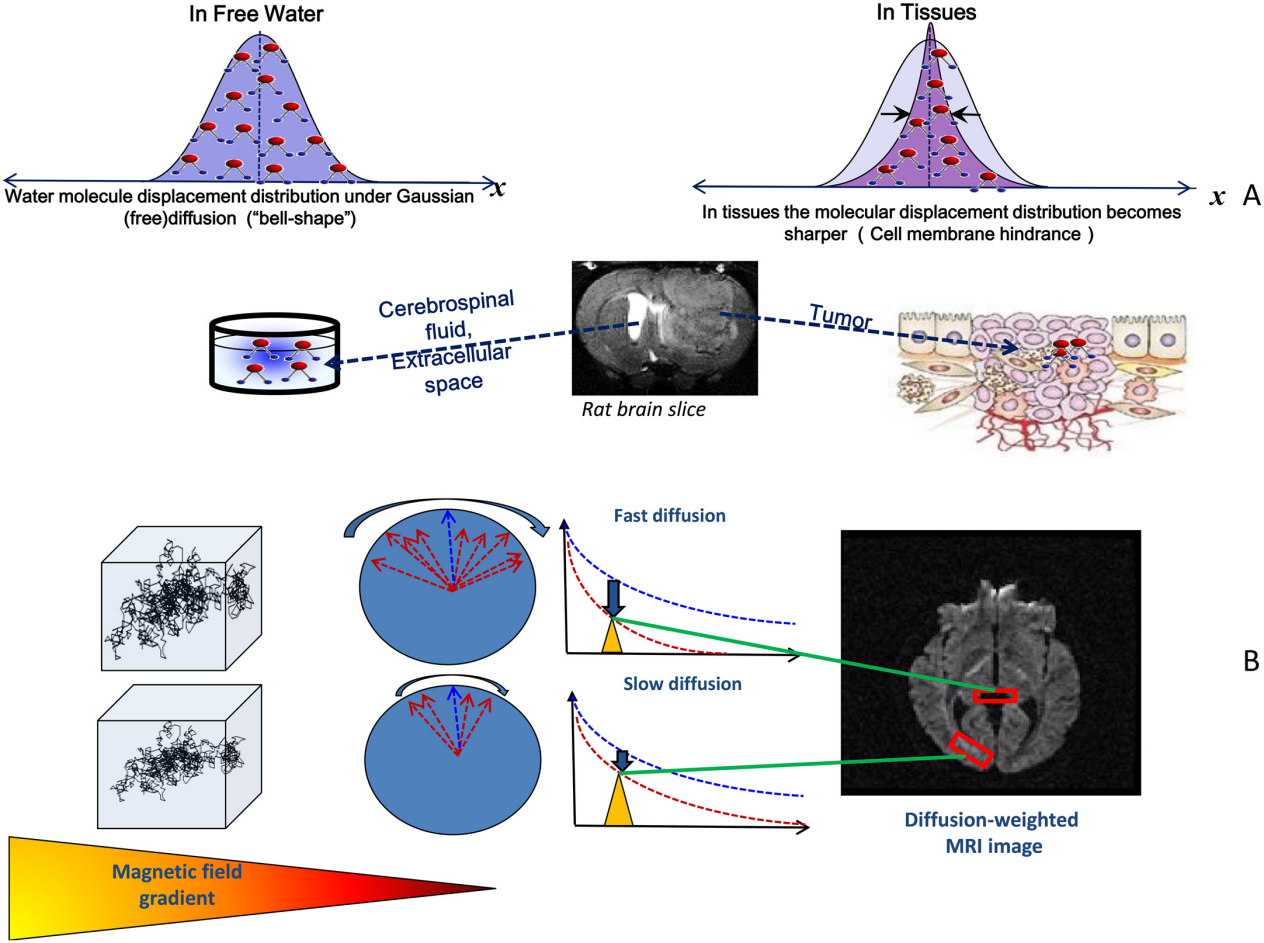
Principles of diffusion MRI. (A) When water is free to diffuse, as in a glass or in brain ventricles containing cerebrospinal fluid (CSF), random water molecular displacement obeys a Gaussian distribution, the width at half height of which gives the diffusion coefficient. In tissues, diffusion is constrained by the presence of molecular and cellular obstacles, so the displacement distribution becomes sharper, especially in tumors where cell density is high. As a result, the diffusion coefficient (width at half height of the displacement distribution) appears reduced compared with free diffusion. (B) In the presence of a magnetic field gradient, the MRI resonant frequency will vary along the direction of the gradient. As a result, the phase of the radiowaves emitted by the magnetized hydrogen nuclei of water molecules contained in a voxel (box representing the image elementary volume) will vary (red arrows) compared to otherwise static nuclei (blue arrow), depending on their displacement behavior. For the diffusion-driven random displacements, the average phase shift is zero but exhibits a distribution that is wider for water nuclei experiencing large displacements (fast diffusion, as in CSF, top) than for those experiencing small displacements (slow diffusion, as in white matter brain tissue, bottom). Considering the very large number of water molecules present in each image voxel, each with its own random displacement history, this phase distribution results in an attenuation of the MRI signal amplitude due to phase interference, and the MRI signal (red curve) decays faster than in the absence of diffusion (blue curve). This attenuation is larger in voxels where water movement is fast, and hence where diffusion is high, and vice versa. The MRI images obtained at a given time (yellow triangle) are then “diffusion weighted”: regions of slow diffusion appear in “white” and those with fast diffusion in “black.” Quantitative maps of the apparent diffusion coefficient can be calculated based on this differential signal attenuation.

The self-diffusion coefficient of free water is around 3.0 x 10^−9^ m^2^/s at 37°C, which translates to about 32% of the molecules having reached at least 17 μm during 50 milliseconds, while only 5% of them have travelled over distances greater than 34 μm. In biological tissues, however, the actual diffusion distance is reduced to a few micrometers—the right scale to explore tissue structure—because of the presence of obstacles. Diffusion-driven displacements of water molecules are encoded in the MRI signal by spatial and temporal variation of the magnetic field (see [2] for a review of the history and the principles of diffusion MRI) generated by magnetic field gradient pulses. The overall effect of diffusion in the presence of those gradient pulses is a signal attenuation, and the MRI signal becomes “diffusion-weighted.” The signal attenuation is more pronounced when using large gradient pulses and when diffusion is fast (Fig 1).

Another important consideration is that in biological tissues diffusion is not free but hindered by many obstacles such as cell membranes, fibers or macromolecules, and macromolecular crowding. Einstein’s diffusion equation, on which diffusion MRI is based, assumes “free” diffusion, as in a glass of water, and the distribution of diffusion-driven molecular displacements obeys a Gaussian law. In biological tissues, the molecular displacement distribution deviates from a Gaussian law, and, thus, the diffusion effect on the MRI signal is no longer adequately described by Einstein’s equation (Fig 1). Consequently, the diffusion coefficient derived from diffusion-weighted images is no longer the free diffusion coefficient of water. The observation of non-Gaussian diffusion and the related modeling of diffusion effects was investigated by pioneers such as Stejskal and Tanner (see [3] for a review), well before the advent of MRI, but this issue remains a complex and hot topic of investigation today for diffusion MRI [4].

The “apparent diffusion coefficient” (ADC) concept was introduced along with the diffusion MRI concept [5] to avoid the difficulties of non-Gaussian diffusion and facilitate clinical application of the technique. The idea was to use Einstein’s equation to model diffusion MRI signals as if water diffusion was Gaussian but to describe, instead, the results as an ADC, to emphasize that results differ from a free diffusion coefficient. This ADC concept proved extremely powerful and is widely used today [6]. Indeed, non-Gaussian diffusion effects at the microscopic level, reflecting the interaction of water molecules with tissue elements, are intrinsically present in the ADC, explaining its high sensitivity to pathologic or physiologic conditions encountered in tissues. The ADC also depends on the measurement conditions, especially the intensity and the time profiles of the gradient pulses, which set the time window to observe diffusion. With long diffusion times, water molecules have a higher probability to interact with tissue microscopic features than when short diffusion times are used, leading to less signal attenuation and, thus, to a smaller ADC at long diffusion times [7].

Characterizing the non-Gaussian diffusion, MRI signal behavior can provide extremely valuable information on tissue structure. A variety of models have been proposed to handle this non-Gaussian behavior. With such models, new parameters have emerged beyond the ADC. The polynomial model, especially, provides a measure of the degree of deviation of diffusion from a Gaussian law—known as kurtosis—that has great potential to characterize pathological or physiological conditions, although it only gives “empirical” information on the degree of non-Gaussian diffusion and not on the genuine tissue microstructure. The kurtosis parameter is currently under investigation to evaluate a variety of diseases, such as cerebral infarction [8], liver fibrosis [9], and tumors [10–12]. Other, more “explanatory,” models have been designed to provide more insightful information on tissue features, mainly in the brain, such as the axon diameter in white matter (CHARMED [composite hindered and restricted model of diffusion] and AxCaliber models [13,14]) or the gray matter neurite distribution (neurite orientation dispersion and density imaging [NODDI] model [15]); however, these extremely sophisticated models make important assumptions about the underlying tissue structure and are yet to be validated for a variety of clinical conditions.

## Applications of Diffusion MRI

### Acute Brain Ischemia

**Fig 2:**
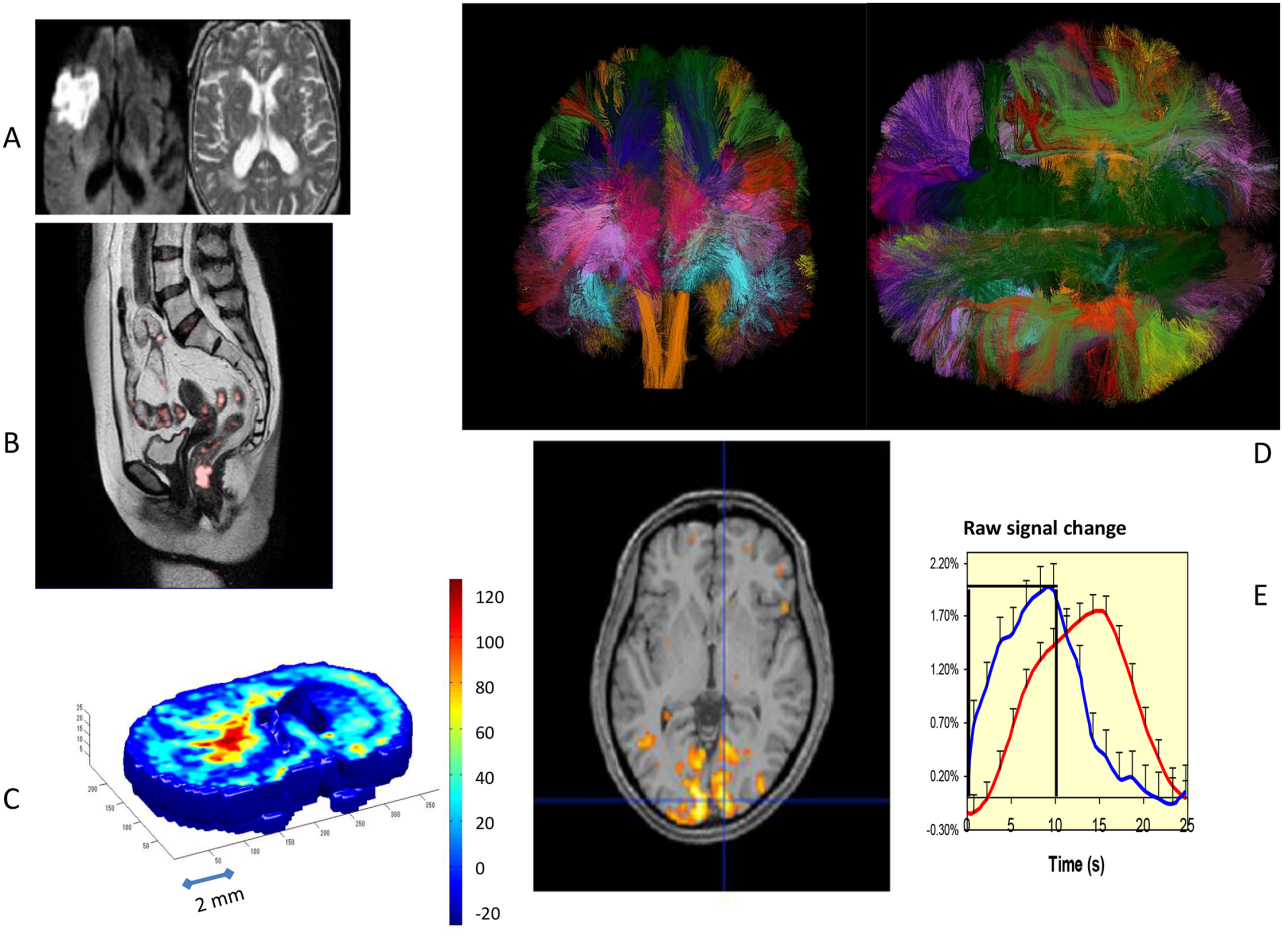
Main applications of water diffusion MRI. (A) Acute stroke. The diffusion-weighted image (bottom) clearly shows a bright signal corresponding to a drop in water diffusion resulting from cell swelling (cytotoxic edema) in the tissue undergoing acute ischemia. The conventional MRI image (top) shows no abnormal feature. (B) Pelvic cancer. Water diffusion is usually reduced in malignant tissues compared to normal tissues because of the underlying cell proliferation in the tumor. Areas with reduced diffusion are shown in pink (a primary cancer in the rectum with several metastases). (C) Main applications of water diffusion MRI: rat brain 9L glioma model. Diffusion MRI is widely used for preclinical research, for instance, to evaluate the effects of new therapies on cancers. Here, in a composite image of the ADC and the kurtosis parameter (arbitrary color scale from blue for normal tissue to red for highly proliferating tissue), the developing tumor appears in red as an area of low diffusion and high kurtosis, reflecting diffusion hindrance from cell proliferation. (D) Main applications of water diffusion MRI: brain connectivity. Water diffusion in brain white matter fibers is anisotropic, i.e., faster in the direction of the fibers. By measuring water diffusion in many directions, the orientations of the whiter matter bundles can be determined at each brain location. Algorithms then identify bundles, which are represented with arbitrary colors (courtesy of C. Poupon, CONNECT/NeuroSpin). (E) Main applications of water diffusion MRI: diffusion functional neuroimaging. Water diffusion decreases during activation of neural tissue (here the primary visual cortex was stimulated by a flickering checkerboard for 10 seconds). The time course of the diffusion MRI responses (blue) appears much faster than the usual blood oxygenation level-dependent (BOLD) response (red) both at onset and offset. The BOLD response results from a local increase in blood flow. The diffusion response might reflect more directly cellular events occurring in the neural tissue upon activation, such as cellular swelling.

Around 1990, Michael Moseley at the University of California at San Francisco made an unexpected but crucial discovery [16]: water diffusion dropped significantly (30%–50%) during the very early phase of acute brain ischemia in cats. This finding tremendously boosted diffusion MRI, then still essentially a pure research tool, by attracting clinicians and convincing manufacturers to implement it on their systems. This move to the clinical field became possible with the advent of echo-planar imaging (EPI) MRI. With this technique, MRI images could be acquired as a snapshot in just a fraction of a second, virtually freezing patient motion [17], allowing patients suffering an acute stroke (within 3–6 hours of onset) to be scanned in just a few minutes. The results immediately and directly confirmed Moseley’s observations on cat brains: water diffusion (ADC) decreased in the infarcted areas where dying brain cells undergo swelling through cytotoxic edema, clearly highlighting those areas as bright spots, whereas standard MRI images (as well as computerized-tomography [CT] scans) usually do not show any clear sign of abnormality for hours, well beyond the therapeutic window (Fig 2A) [18]. Several hypotheses have been proposed to explain this sharp, counterintuitive decrease in water diffusion, but the exact mechanisms linking the ADC decrease with cell swelling still remain today to be clarified [3].

Around the same time, Genentech was developing an intravenous recombinant tissue plasminogen activator (rtPA) drug aimed at thrombolytic therapy for acute stroke patients. Clearly, diffusion MRI could not have arrived at a better time, and this coincidental match became a milestone in the history of the management of acute stroke patients. Indeed, with its exquisite sensitivity compared to other imaging modalities, such as X-ray CT, diffusion MRI provides patients with the opportunity to receive suitable treatment at a stage when brain tissue might still be salvageable, thus avoiding them terrible handicaps. Diffusion MRI has made significant changes to the management of stroke patients, not only to customize therapeutic approaches (pharmacological or interventional) for individual patients [19], but also to monitor patient progress on an objective basis (in both the acute and the chronic phase [20]) and to predict clinical outcome [19,21–23].

### Cancer

Diffusion MRI is also rapidly becoming the method of choice to manage cancer patients. Diffusion MRI is increasingly being used in place of fluoro-deoxyglucose positron emission tomography (FDG-PET), which shows areas with increased glucose metabolism characteristic of cancer. Diffusion MRI has the advantages that it does not use ionizing radiation and does not require injection of any tracer, also offering a superior spatial resolution to that of FDG-PET (PET scanners are nowadays available as hybrid systems including X-ray CT or MRI to boost spatial resolution). Diffusion MRI provides excellent imaging of the pelvis (Fig 2B), where FDG-PET may be of limited value because of the accumulation of the tracer radioactivity in the bladder and artifacts related to the pelvic bones. Moreover, FDG uptake can be nonspecific and may occur in inflammation, whereas diffusion MRI tends to be more specific to cell proliferation. Nonetheless, FDG-PET may remain the method of choice for organs that are difficult to image with MRI, such as lymph nodes or lungs.

Water diffusion slows down when tumor cellularity increases [12,24–27]: a large ADC decrease is usually associated with a high degree of malignancy [28,29], whereas high ADC values might predict a poor response to therapy [30–32]. The kurtosis parameter may even be more specific than the ADC (Fig 2C) [33]. Diffusion MRI appears very promising not only to detect or stage malignant lesions but also to follow disease progression or effects of treatments on an individual patient basis [34]. Major applications are for breast, prostate, liver, kidney, and lymphoma [35]. Diffusion MRI has a high potential as a clinical biomarker for the assessment of new drug development and clinical practice [35], but several technical issues remain to be addressed—for instance, to suppress from the images fat, which also exhibits very low ADCs [36]. The relationship between the diffusion parameters and the underlying tissue structure at the microscopic level and changes induced by therapy also remains to be investigated.

### Wiring of the Brain

One important consideration in the application of diffusion MRI is that diffusion, although a three-dimensional process, is only measured in one dimension at a time, determined by the orientation in space of the gradient pulses. Most often diffusion is “isotropic” (the same in all directions), so the spatial orientation does not matter. In some tissues, however, diffusion may be “anisotropic,” which means that diffusion effects strongly depend on the gradient pulse directions. Indeed, around 1990, water diffusion in white matter fibers of the brain was found to be anisotropic: diffusion was fast in the direction of the fibers and slower perpendicularly to them [16]. Anisotropic diffusion cannot be correctly handled by simply measuring three diffusion coefficients along three perpendicular axes but requires switching to a mathematical tensor formalism. With “diffusion tensor imaging” (DTI) [37], one can obtain for each image voxel information on the directions along which diffusion is fastest or the slowest, corresponding in general to the directions parallel or perpendicular to the tissue fibers, respectively.

After this discovery, it was soon suggested that this feature could be used to determine and map the orientation of white matter fibers in the brain, assuming the direction of the fibers was parallel to the direction with the fastest diffusion. The first attempt was very crude, with diffusion being measured along two directions only, but the concept of white matter fiber orientation color mapping was born and a proof of principle provided [38]. Progress from those basic images to the gorgeous fiber tract 3-D displays that now make up the covers of journals and anatomy textbooks (Fig 2D) implied a big step, which was made possible with DTI. Algorithms were developed at the end of the 1990s to connect those voxels containing white matter fiber bundles together [39–41], resulting in the world’s first 3-D representations of the fiber bundles (with very colorful representations of the “white” matter) within the human brain. The potential of diffusion MRI to probe human brain connectivity has attracted worldwide interest and is now widely used in clinical practice. Recent results from the European FP7 CONNECT project [42] and the Human Connectome Project [43] have clearly underlined the enormous potential of this approach, yielding insight into how brain connections underlie function and opening up new lines of inquiry for human neuroscience and brain dysfunction in aging, mental health disorders, addiction, and neurological disease [2]. DTI is commonly used to investigate white matter disorders and has also revealed faulty brain connections linked to dyslexia, schizophrenia, autism, bipolar or anxiety disorders, suggesting that temporal synchronization in brain processing may be altered in patients with those disorders [44]. Technical progress to boost gradient pulse systems performance is awaited [43], as it might significantly improve the resolution of the visualized tracts, up to the junction with the cortex.

### Neuronal Activation and Functional Neuroimaging

Functional neuroimaging has become an essential approach to study the brain and the mind. In the past, PET and, nowadays, functional MRI (fMRI, based on the BOLD contrast mechanism [45]) have relied on the principle that neuronal activation and blood flow are coupled through metabolism, and brain activation can be imaged indirectly through variations in local blood flow. With diffusion MRI, a new paradigm has emerged whereby we can look at brain activity through the observation of water molecular diffusion. Water diffusion is, indeed, modulated by brain activity [46]: the diffusion signal response is characterized by a sharp peak, faster than the indirect hemodynamic response (i.e., increase in blood flow) observed with BOLD fMRI (Fig 2E) [47]. The diffusion response persists after inhibition of neurovascular coupling (which suppresses the BOLD fMRI response) and shares the features of the underlying neuronal response [48]. This “diffusion fMRI” approach is, thus, more directly linked to neuronal function than blood-flow-based approaches, a paradigm shift in the way we visualize brain activity. Indeed, diffusion MRI is exquisitely sensitive to changes in the neural tissue microstructure. Activation-driven changes in tissue microstructure, such as cell swelling, have been reported in many instances in the literature. As cell swelling in tissues has been shown to result in a decrease in water diffusion detectable by MRI, we have hypothesized that the slowing of diffusion observed during neuronal activation might reflect neuronal cell swelling [46] occurring within the activated cortical ribbon (probably at the level of the dendrites and their spines). Based on this “electromechanical coupling,” neurons might perhaps be seen as piezoelectric sensors: variations in cell shape should, in return, induce cell depolarization, potentially allowing a very fast, nonsynaptic transmission mechanism within neural clusters of the cortical ribbon. Indeed, dynamic changes in neuron structure (especially of dendritic spines) are now thought to play an important role in the function of such cell clusters, as envisioned by Crick [49] and even Ramon y Cajal [50]): “The state of activity would correspond to the swelling and elongation of the [dendritic] spines, and the resting state (sleep or inactivity) to their retraction.” Recent work has demonstrated that local neural activation could be modulated by the mechanical pressure induced by focused ultrasound beams produced by transducers placed around the skull [51]. Diffusion MRI has the potential to address such questions and might allow us to further our understanding of the biophysical mechanisms associated with neuronal activation and the role of water in such mechanisms, up to the mechanisms of consciousness [52].

## Challenges for the Future

Diffusion MRI has the potential to provide information on the cellular organization of tissues noninvasively and in vivo. Still, the mechanisms governing the diffusion of water in tissues, notably in the brain, remain unclear. A more profound understanding of such mechanisms will be necessary to exploit fully the potential of the technique and to obtain information directly on tissue microstructure. Tissues are complex, organized structures in which diffusion largely differs from the Brownian motion modeled by Einstein. A unified model has still to be found to explain tissue water diffusion. Besides empirical models, which are merely used to describe the diffusion MRI signal behavior, most models have focused on mechanistic features of tissues (e.g., compartments such as the intra- and extracellular compartments and physical obstacles, such as fibers and cell membranes). Among cellular components, there is growing evidence that membranes, even if they are permeable, are likely to be the main feature that hinders water diffusion. Water diffusion is modulated by cell size (decreasing with cell swelling, as observed in stroke or perhaps neuronal activation), cell density (falling with the increased membrane content), and cell/membrane orientation (diffusion anisotropy in white matter fibers), features which are all impacting cell membranes. Explanations of the factors that limit diffusion in tissues have remained often qualitative, not to say imaginative. To understand better, some physical modeling will be essential.

Recent data on the physical properties of water and on the status of water in biological tissues suggest that the biophysical mechanisms of water diffusion in tissues may not result solely from mechanistic tissue features but may also result from functional features at the molecular level. The presence (or the amount) of structured water in cells is itself a subject of great controversy among physicists and biologists. We have probably largely underestimated the importance of water in biology, from protein and membrane dynamics to cell physiology. Besides protein-bounding through hydrogen bounds, water also forms molecular networks whose properties, including diffusion, may be altered in the vicinity of charged cellular or intracellular membranes. Given the large surface area/volume ratio of most cells, such a membrane-interacting water network probably constitutes an important fraction of tissue water. Hence, any fluctuation in the shape, size, or density of cells (or subcellular components) would induce large variations in the membrane surface area, which could impact the diffusion MRI signal. Indeed, neural tissues are not static but are continuously undergoing dynamic changes.

**Fig 3:**
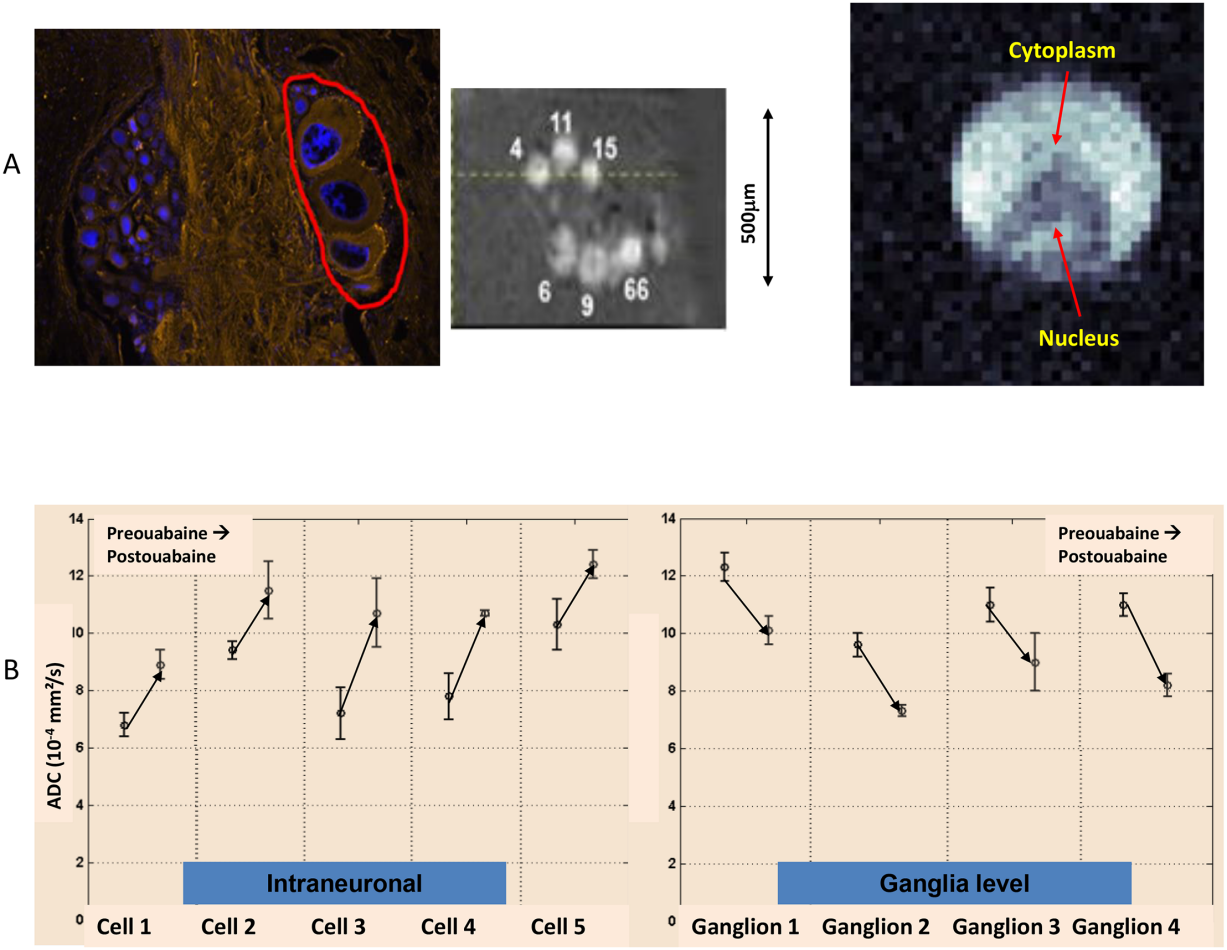
Diffusion MRI in single neurons and buccal ganglia of *Aplysia californica* obtained with a 17.2 tesla MRI system. (A) Image of a single neuron at 25 μm resolution. Left: the approximate region of cell bodies chosen for the diffusion measurements are indicated by the red outline on a fixed and immunostained ganglion slice (nuclei are labeled in blue and the cytoskeleton and neurites in orange). Middle: MRI image of this selected region; right: MRI image of a single neuron within the selected region (see reference [53] for details). (B) ADC measurements in the soma (left) and in the ganglia (right), pre- and poststimulation with oubain, an inhibitor of the plasma membrane Na+/K+ ATPase, which causes cell swelling. The ADC increases in the soma by 30% and decreases in the ganglion tissue by 18% upon treatment with the inhibitor. This discrepancy in diffusion behavior suggests the importance of cell membranes for the ADC measured at tissue level (where a hypothetical layer of slowly diffusing water molecules bound to membranes would increase in size upon cell swelling and membrane surface expansion). Courtesy of I. Jelescu and L. Ciobanu, NeuroSpin.

Using MRI scanners operating at an extremely high magnetic field (such as 17.2 teslas), one can boost the image resolution to the level of single neurons, which can pin down diffusion mechanisms at the cellular level [53]. By using this approach, water diffusion has been measured inside the soma of single neurons and in the region of cell bodies of the buccal ganglia of Aplysia upon exposure to ouabain, which inhibits Na^+^/K^+^ pumps. Results showed an increase in water diffusion inside isolated neurons but a decrease at tissue level (Fig 3) [53]. These contradictory findings cannot be explained with current “mechanistic” tissue diffusion models. The scenario involving a layer of water molecules bound to the inflating cell membrane surface has been proposed to reconcile this apparent discrepancy. To make progress, much stronger gradient hardware than is currently available on clinical MRI scanners will be needed to measure short diffusion times. By acquiring water diffusion data on different time scales, it may be possible to modulate the amount of water–membrane interactions that contribute to the diffusion MRI signal, a step to reveal interesting features of the brain tissues.

Another appealing avenue is to look at molecular species that are clearly compartmentalized, such as cell metabolites, by using diffusion MR spectroscopy. Progress has been made in this direction, but further advances will also require powerful gradient pulse systems to observe large molecules with slow diffusion coefficients, as their molecular displacements remain much smaller than those of water molecules given their size. While gradient strengths of 1–2 teslas/m are not uncommon on animal MRI systems, strengths of 0.1 tesla/m or even 0.3 tesla/m have been reached on prototype clinical MRI scanners made for the United States Human Connectome Project [43], leading to a sensitivity to diffusion about 100 times higher than in 1986, when diffusion MRI was introduced.

With such powerful gradient systems, the high spatial resolution available with MRI scanners operating at ultra-high magnetic field (above 10 teslas) and the computational power of large computers, one may dream of obtaining in vivo important information on the spatial organization of the cortical components (neurons, glial cells, dendrites, or spines) of the human brain, which are known to vary dramatically across the brain cortex, as first reported by Brodmann in 1908 through the histological observation of half of a dead brain. Indeed, the “Brodmann areas” [54] are deemed to be associated each with specific brain functions, and most functional neuroimaging studies have relied on the cytoarchitectonic classification of Brodmann to report the location of activated regions along the brain cortex. To do so, standardized atlases of the brain and the Brodmann areas have been built, based on the 1908 seminal observation, to which fMRI images are registered and grossly matched, thus ignoring individual variations in brain microstructure. Diffusion MRI (based on water or on cell specific metabolites, such as N-acetyl-aspartate or neurotransmitters) has the potential to provide specific information on brain cytoarchitectony of single individuals. From this three-dimensional spatial organization of cell clusters along the brain cortex (or in basal ganglia) could perhaps emerge some sort of generic neural code supporting a set of basic neural functions, in a way similar to that which supports the genetic code from the very specific spatial arrangement of nucleotides along DNA. Higher-order functions would result from a combination of those elementary neural functions through dynamic connections between adjacent or distant neuron clusters. What better approach than diffusion MRI could give us, in vivo and noninvasively, especially in the human brain, exquisite information on both cytoarchitectony and connections?

Finally, while MRI is merely a means, although very convenient, to visualize the diffusion of water or other molecules in tissues, one should not forget that diffusion is an ubiquitous process that can be investigated to understand cell physiology and life. All biological processes—DNA replication, RNA transcription, protein translation, protein and enzyme activity, cross membrane transport, etc.—require molecules to interact. Before molecules can interact, however, they must come into molecularly close proximity. Diffusion appears to be the universal process through which this occurs. In a sense, diffusion rates set the speed limit for life, just as light sets the limit of speed in the physical world. Diffusion MRI is a great tool at our fingertips to explore this process.

## Abbreviations

ADC: apparent diffusion coefficient
BOLD: blood oxygenation level dependent
CHARMED: composite hindered and restricted model of diffusion
CSF: cerebrospinal fluid
CT: computerized tomography
DTI: diffusion tensor imaging
EPI: echo-planar imaging
FDG-PET: fluoro-deoxyglucose positron emission tomography
fMRI: functional MRI
MRI: magnetic resonance imaging
NODDI: neurite orientation dispersion and density imaging
PET: positron emission tomography
rtPA: recombinant tissue plasminogen activator
