# Correction: Diffusion Magnetic Resonance Imaging: What Water Tells Us about Biological Tissues

**DOI:** 10.1371/journal.pbio.1002246

**Published:** 2015-09-03

**Authors:** Denis Le Bihan, Mami Iima

Some errors were introduced in the ordering of the references in the published paper. References 1–5 were listed out of order and therefore also caused errors within the in-text citations, as detailed below:

1. In the first paragraph of the section titled ‘Principles of Diffusion MRI and Key Concepts’, the third sentence should have cited reference 2, instead of 1. The correct sentence should read:

Unexpectedly, diffusion (a visible phenomenon) was linked by Einstein to Brownian motion in the context of the theory of heat to prove the existence of (invisible) atoms and molecules [2].

2. In the second paragraph, titled ‘Principles of Diffusion MRI and Key Concepts’, the third to last sentence should have cited reference 3, instead of 2. The correct sentence should read:

Diffusion-driven displacements of water molecules are encoded in the MRI signal by spatial and temporal variation of the magnetic field (see [3] for a review of the history and the principles of diffusion MRI) generated by magnetic field gradient pulses.

3. In the second paragraph of the section titled ‘Principles of Diffusion MRI and Key Concepts’, the last sentence should have cited references 4 and 5 respectively, rather than 3 and 4. The correct sentence should read:

The observation of non-Gaussian diffusion and the related modeling of diffusion effects was investigated by pioneers such as Stejskal and Tanner (see [4] for a review), well before the advent of MRI, but this issue remains a complex and hot topic of investigation today for diffusion MRI [5].

4. In the third paragraph of the section titles ‘Principles of Diffusion MRI and Key Concepts’, the first sentence should have cited reference 1 rather than 5. The correct sentence should read:

The “apparent diffusion coefficient” (ADC) concept was introduced along with the diffusion MRI concept [1] to avoid the difficulties of non-Gaussian diffusion and facilitate clinical application of the technique.

5. In the first paragraph of the section titled ‘Applications of Diffusion MRI; Acute Brain Ischemia’ the last sentence cited reference 3 rather than 4. The correct sentence should read:

Several hypotheses have been proposed to explain this sharp, counterintuitive decrease in water diffusion, but the exact mechanisms linking the ADC decrease with cell swelling still remain today to be clarified [4].

6. In the section titled ‘Wiring of the Brain’, the third from last sentence cited reference 2 rather than 3. The correct sentence should read:

The potential of diffusion MRI to probe human brain connectivity has attracted worldwide interest and is now widely used in clinical practice. Recent results from the European FP7 CONNECT project [42] and the Human Connectome Project [43] have clearly underlined the enormous potential of this approach, yielding insight into how brain connections underlie function and opening up new lines of inquiry for human neuroscience and brain dysfunction in aging, mental health disorders, addiction, and neurological disease [3].

The full corrected list of references is also provided here:

1. Le Bihan D, Breton E, Lallemand D, Grenier P, Cabanis E, Laval-Jeantet M. MR imaging of intravoxel incoherent motions: application to diffusion and perfusion in neurologic disorders. Radiology. 1986; 161 (2):401–7. doi: 10.1148/radiology.161.2.3763909 PMID: 3763909
2. Einstein A. Investigations on the Theory of the Brownian Movement: Courier Dover Publications; 1956.
3. Le Bihan D, Johansen-Berg H. Diffusion MRI at 25: exploring brain tissue structure and function. Neuroimage. 2012; 61(2):324–41. doi: 10.1016/j.neuroimage.2011.11.006 PMID: 22120012
4. Le Bihan D. The 'wet mind': water and functional neuroimaging. Physics in Medicine and Biology. 2007; 52(7):R57–90. doi: 10.1088/0031-9155/52/7/r02 PMID: 17374909
5. Yablonskiy DA, Sukstanskii AL. Theoretical models of the diffusion weighted MR signal. NMR in biomedicine. 2010; 23(7):661–81. doi: 10.1002/nbm.1520 PMID: 20886562
6. Le Bihan D. Apparent diffusion coefficient and beyond: what diffusion MR imaging can tell us about tissue structure. Radiology. 2013; 268(2):318–22. doi: 10.1148/radiol.13130420 PMID: 23882093
7. Pyatigorskaya N, Le Bihan D, Reynaud O, Ciobanu L. Relationship between the diffusion time and the diffusion MRI signal observed at 17.2 tesla in the healthy rat brain cortex. Magnetic Resonance in Medicine. 2014; 72(2):492–500. doi: 10.1002/mrm.24921 PMID: 24022863
8. Grinberg F, Farrher E, Ciobanu L, Geffroy F, Le Bihan D, Shah NJ. Non-Gaussian Diffusion Imaging for Enhanced Contrast of Brain Tissue Affected by Ischemic Stroke. PLoS ONE. 2014; 9(2):e89225. doi:10.1371/journal.pone.0089225 PMID: 24586610
9. Anderson SW, Barry B, Soto J, Ozonoff A, O'Brien M, Jara H. Characterizing non‐gaussian, high bvalue diffusion in liver fibrosis: Stretched exponential and diffusional kurtosis modeling. J Magn Reson Imaging. 2014; 39(4):827–34. doi: 10.1002/jmri.24234 PMID: 24259401
10. Yuan J, Yeung DKW, Mok GS, Bhatia KS, Wang Y-XJ, Ahuja AT, et al. Non-Gaussian Analysis of Diffusion Weighted Imaging in Head and Neck at 3T: A Pilot Study in Patients with Nasopharyngeal Carcinoma. PLoS ONE. 2014; 9(1):e87024. doi: 10.1371/journal.pone.0087024 PMID: 24466318
11. Iima M, Yano K, Kataoka M, Umehana M, Murata K, Kanao S, et al. Quantitative non-gaussian diffusion and intravoxel incoherent motion magnetic resonance imaging: differentiation of malignant and benign breast lesions. Invest Radiol. 2015; 50(4):205–11. doi: 10.1097/RLI.0000000000000094 PMID: 25260092
12. Iima M, Reynaud O, Tsurugizawa T, Ciobanu L, Li J-R, Geffroy F, et al. Characterization of Glioma Microcirculation and Tissue Features Using Intravoxel Incoherent Motion Magnetic Resonance Imaging in a Rat Brain Model. Invest Radiol. 2014; 49(7):485–90. doi: 10.1097/RLI.0000000000000040 PMID: 24619211
13. Assaf Y, Basser PJ. Composite hindered and restricted model of diffusion (CHARMED) MR imaging of the human brain. Neuroimage. 2005; 27(1):48–58. doi: 10.1016/j.neuroimage.2005.03.042 PMID: 15979342
14. Assaf Y, Blumenfeld‐Katzir T, Yovel Y, Basser PJ. AxCaliber: a method for measuring axon diameter distribution from diffusion MRI. Magnetic Resonance in Medicine. 2008; 59(6):1347–54. doi: 10.1002/mrm.21577 PMID: 18506799
15. Zhang H, Schneider T, Wheeler-Kingshott CA, Alexander DC. NODDI: Practical in vivo neurite orientation dispersion and density imaging of the human brain. Neuroimage. 2012; 61(4):1000–16. doi: 10.1016/j.neuroimage.2012.03.072 PMID: 22484410
16. Moseley M, Cohen Y, Mintorovitch J, Chileuitt L, Shimizu H, Kucharczyk J, et al. Early detection of regional cerebral ischemia in cats: comparison of diffusion and T2‐weighted MRI and spectroscopy. Magnetic Resonance in Medicine. 1990; 14(2):330–46. doi: 10.1002/mrm.1910140218 PMID: 2345513
17. Turner R, Le Bihan D, Maier J, Vavrek R, Hedges LK, Pekar J. Echo-planar imaging of intravoxel incoherent motion. Radiology. 1990; 177(2):407–14. doi: 10.1148/radiology.177.2.2217777 PMID: 2217777
18. Warach S, Chien D, Li W, Ronthal M, Edelman RR. Fast magnetic resonance diffusion-weighted imaging of acute human stroke. Neurology. 1992; 42(9):1717–23. doi: 10.1212/wnl.42.9.1717 PMID: 1513459
19. Warach S, Boska M, Welch KM. Pitfalls and potential of clinical diffusion-weighted MR imaging in acute stroke. Stroke; a journal of cerebral circulation. 1997; 28(3):481–2. PMID: 9056599
20. Warach S, Dashe JF, Edelman RR. Clinical outcome in ischemic stroke predicted by early diffusion weighted and perfusion magnetic resonance imaging: a preliminary analysis. Journal of Cerebral Blood Flow and Metabolism. 1996; 16(1):53–9. doi: 10.1002/mrm.1910390605 PMID: 8530555
21. Dreher W, Kuhn B, Gyngell ML, Busch E, Niendorf T, Hossmann KA, et al. Temporal and regional changes during focal ischemia in rat brain studied by proton spectroscopic imaging and quantitative diffusion NMR imaging. Magnetic Resonance in Medicine 1998; 39(6):878–88. doi: 10.1002/mrm.1910390605 PMID: 9621911
22. Gonzalez RG, Schaefer PW, Buonanno FS, Schwamm LH, Budzik RF, Rordorf G, et al. Diffusion weighted MR imaging: diagnostic accuracy in patients imaged within 6 hours of stroke symptom onset. Radiology. 1999; 210(1):155–62. doi: 10.1148/radiology.210.1.r99ja02155 PMID: 9885601
23. Lovblad KO, Baird AE, Schlaug G, Benfield A, Siewert B, Voetsch B, et al. Ischemic lesion volumes in acute stroke by diffusion-weighted magnetic resonance imaging correlate with clinical outcome. Annals of neurology. 1997; 42(2):164–70. doi: 10.1002/ana.410420206 PMID: 9266725
24. Guo Y, Cai YQ, Cai ZL, Gao YG, An NY, Ma L, et al. Differentiation of clinically benign and malignant breast lesions using diffusion‐weighted imaging. J Magn Reson Imaging. 2002; 16 (2):172–8. doi: 10.1002/jmri.10140 PMID: 12203765
25. Humphries PD, Sebire NJ, Siegel MJ, Olsen ØE. Tumors in Pediatric Patients at Diffusion-weighted MR Imaging: Apparent Diffusion Coefficient and Tumor Cellularity Radiology. 2007; 245(3):848–54. doi: 10.1148/radiol.2452061535 PMID: 17951348
26. Sugahara T, Korogi Y, Kochi M, Ikushima I, Shigematu Y, Hirai T, et al. Usefulness of diffusion weighted MRI with echo‐planar technique in the evaluation of cellularity in gliomas. J Magn Reson Imaging. 1999; 9(1):53–60. doi: 10.1002/(sici)1522-2586(199901)9:1<53::aid-jmri7>3.0.co;2–2 PMID: 10030650
27. Fan G, Zang P, Jing F, Wu Z, Guo Q. Usefulness of Diffusion/Perfusion-weighted MRI in Rat Gliomas: Correlation with Histopathology. Academic Radiology. 2005; 12(5):640–51. doi: 10.1016/j.acra.2005.01.024 PMID: 15866139
28. Nakajo M, Kajiya Y, Kaneko T, Kaneko Y, Takasaki T, Tani A, et al. FDG PET/CT and diffusion weighted imaging for breast cancer: prognostic value of maximum standardized uptake values and apparent diffusion coefficient values of the primary lesion. European Journal of Nuclear Medicine and Molecular Imaging. 2010; 37(11):2011–20. doi: 10.1007/s00259-010-1529-7 PMID: 20607535
29. Turkbey B, Shah VP, Pang Y, Bernardo M, Xu S, Kruecker J, et al. Is apparent diffusion coefficient associated with clinical risk scores for prostate cancers that are visible on 3-T MR images? Radiology.2011; 258(2):488–95. doi: 10.1148/radiol.10100667 PMID: 21177390
30. Cui Y, Zhang X-P, Sun Y-S, Tang L, Shen L. Apparent Diffusion Coefficient: Potential Imaging Biomarker for Prediction and Early Detection of Response to Chemotherapy in Hepatic Metastases Radiology. 2008; 248(3):894–900. doi: 10.1148/radiol.2483071407 PMID: 18710982
31. Koh D-M, Scurr E, Collins D, Kanber B, Norman A, Leach MO, et al. Predicting response of colorectal hepatic metastasis: value of pretreatment apparent diffusion coefficients. American Journal of Roentgenology.2007; 188(4):1001–8. doi: 10.2214/ajr.06.0601 PMID: 17377036
32. Sun Y-S, Zhang X-P, Tang L, Ji J-F, Gu J, Cai Y, et al. Locally Advanced Rectal Carcinoma Treated with Preoperative Chemotherapy and Radiation Therapy: Preliminary Analysis of Diffusion-weighted MR Imaging for Early Detection of Tumor Histopathologic Downstaging Radiology. 2009; 254(1):170–8. doi: 10.1148/radiol.2541082230 PMID: 20019139
33. Filli L, Wurnig M, Nanz D, Luechinger R, Kenkel D, Boss A. Whole-Body Diffusion Kurtosis Imaging: Initial Experience on Non-Gaussian Diffusion in Various Organs. Investigative Radiology. 2014; 49 (12):773–8. doi: 10.1097/RLI.0000000000000082 PMID: 24979203
34. Patterson DM, Padhani AR, Collins DJ. Technology insight: water diffusion MRI—a potential new biomarker of response to cancer therapy. Nature Clinical Practice Oncology. 2008; 5(4):220–33. doi: 10.1038/ncponc1073 PMID: 18301415
35. Padhani AR, Liu G, Mu-Koh D, Chenevert TL, Thoeny HC, Takahara T, et al. Diffusion-weighted magnetic resonance imaging as a cancer biomarker: consensus and recommendations. Neoplasia. 2009;11(2):102–25. PMID: 19186405
36. Takahara T, Imai Y, Yamashita T, Yasuda S, Nasu S, Van Cauteren M. Diffusion weighted whole body imaging with background body signal suppression (DWIBS): technical improvement using free breathing, STIR and high resolution 3D display. Radiation Medicine. 2004; 22(4):275–82. PMID: 15468951
37. Basser PJ, Mattiello J, LeBihan D. MR diffusion tensor spectroscopy and imaging. Biophysical Journal.1994; 66(1):259–67. doi: 10.1016/s0006-3495(94)80775-1 PMID: 8130344
38. Douek P, Turner R, Pekar J, Patronas N, Le Bihan D. MR color mapping of myelin fiber orientation. Journal of Computer Assisted Tomography. 1991; 15(6):923–9. doi: 10.1097/00004728-199111000-00003 PMID: 1939769
39. Mori S, Crain BJ, Chacko VP, van Zijl PC. Three-dimensional tracking of axonal projections in the brain by magnetic resonance imaging. Annals of neurology. 1999; 45(2):265–9. doi: 10.1002/1531-8249(199902)45:2<265::aid-ana21>3.0.co;2–3 PMID: 9989633
40. Conturo TE, Lori NF, Cull TS, Akbudak E, Snyder AZ, Shimony JS, et al. Tracking neuronal fiber pathways in the living human brain. Proc Natl Acad Sci U S A. 1999; 96(18):10422–7. doi: 10.1073/pnas.96.18.10422 PMID: 10468624
41. Poupon C, Clark CA, Frouin V, Regis J, Bloch I, Le Bihan D, et al. Regularization of diffusion-based direction maps for the tracking of brain white matter fascicles. Neuroimage. 2000; 12(2):184–95. doi: 10.1006/nimg.2000.0607 PMID:10913324
42. Assaf Y, Alexander DC, Jones DK, Bizzi A, Behrens TE, Clark CA, et al. The CONNECT project: combining macro-and micro-structure. Neuroimage. 2013; 80:273–82. doi: 10.1016/j.neuroimage.2013.05.055 PMID: 23727318
43. McNab JA, Edlow BL, Witzel T, Huang SY, Bhat H, Heberlein K, et al. The Human Connectome Project and beyond: Initial applications of 300mT/m gradients. Neuroimage. 2013; 80:234–45. doi: 10.1016/j.neuroimage.2013.05.074 PMID: 23711537
44. Shizukuishi T, Abe O, Aoki S. Diffusion tensor imaging analysis for psychiatric disorders. Magnetic Resonance in Medical Sciences. 2013; 12(3):153–9. doi: 10.2463/mrms.2012-0082 PMID: 23857149
45. Ogawa S, Lee T-M, Kay AR, Tank DW. Brain magnetic resonance imaging with contrast dependent on blood oxygenation. Proceedings of the National Academy of Sciences. 1990; 87(24):9868–72. 10.1073/pnas.87.24.9868
46. Le Bihan D, Urayama S-i, Aso T, Hanakawa T, Fukuyama H. Direct and fast detection of neuronal activation in the human brain with diffusion MRI. Proceedings of the National Academy of Sciences. 2006;103(21):8263–8. doi:10.1073/pnas.0600644103
47. Aso T, Urayama S-i, Poupon C, Sawamoto N, Fukuyama H, Le Bihan D. An intrinsic diffusion response function for analyzing diffusion functional MRI time series. Neuroimage. 2009; 47(4):1487–95. doi: 10.1016/j.neuroimage.2009.05.027 PMID: 19450693
48. Tsurugizawa T, Ciobanu L, Le Bihan D. Water diffusion in brain cortex closely tracks underlying neuronal activity. Proceedings of the National Academy of Sciences. 2013; 110(28):11636–41. doi: 10.1073/pnas.1303178110
49. Crick F. Do dendritic spines twitch? Trends in Neurosciences. 1982; 5:44–6. doi: 10.1016/0166-2236(82)90020-0
50. Ramon Y, Cajal S. Textura del Sistema Nervioso del Hombre y de los Vertebrados: Madrid, Nicolas Moya; 1899–1904.
51. Legon W, Sato TF, Opitz A, Mueller J, Barbour A, Williams A, et al. Transcranial focused ultrasound modulates the activity of primary somatosensory cortex in humans. Nature Neuroscience. 2014; 17(2):322–9. doi: 10.1038/nn.3620 PMID: 24413698
52. Pauling L. A Molecular Theory of General Anesthesia is attributed to the formation in the brain of minute hydrate crystals of the clathrate type. Science. 1961; 134(3471):15–21. doi: 10.1126/science.134.3471.15
53. Jelescu IO, Nargeot R, Le Bihan D, Ciobanu L. Highlighting manganese dynamics in the nervous system of Aplysia californica using MEMRI at ultra-high field. Neuroimage. 2013; 76:264–71. doi: 10.1016/j.neuroimage.2013.03.022 PMID: 23523801
54. Zilles K, Amunts K. Centenary of Brodmann's map—conception and fate. Nature Reviews Neuroscience. 2010;11(2):139–45. doi: 10.1038/nrn2776. PMID:20046193
